# The diffuse large B-cell lymphoma – where do we stand now in everyday clinical practice

**DOI:** 10.2478/v10019-012-0002-6

**Published:** 2012-01-02

**Authors:** Brigita Gregoric, Vesna Zadnik, Barbara Jezersek Novakovic

**Affiliations:** 1 Division of Medical Oncology and; 2 Department of Epidemiology, Institute of Oncology Ljubljana, Ljubljana, Slovenia

**Keywords:** diffuse large B-cell lymphoma, R-CHOP, treatment result, routine treatments

## Abstract

**Background:**

Due to superior results observed with the addition of rituximab into treatment of patients with the diffuse large B-cell lymphoma (DLBCL),the R-CHOP (rituximab, cyclophosphamide, doxorubicin, vincristine, and prednisolone) regimen and its variants became the standard initial treatment of these patients. However, the treatment recommendations are based on results of clinical studies while the conditions of routine treatment are far different from the ones in clinical studies. The aim of this retrospective study was therefore to compare the treatment results of routinely treated patients with the DLBCL to results reported by some larger studies.

**Patients and methods:**

Two hundred and ninety five patients with the DLBCL were treated between 2004 and 2008 according to the then protocol with R-CHOP or R-CHOP-like regimens at the Institute of Oncology Ljubljana. Treatment response was evaluated according to Cheson’s criteria and the disease-free and overall survival by means of Kaplan Meier survival curves.

**Results:**

Response to treatment in our evaluation diverged from the reported one predominately in the low risk group (international prognostic index [IPI] categorisation) and in the very good prognosis group (revised international prognostic index (R-IPI) categorisation). The determined complete response (CR) rates in other IPI and R-IPI groups were generally within expectations. Also in the disease-free survival the largest discrepancy occurred in the low-risk patient group (3 year disease-free survival rate of 75%) and in the very good prognosis group (4 year disease-free survival rate of 59%). In all other IPI risk groups, the disease-free survival at 3 years (low intermediate risk 76%, high intermediate risk group 57%, and high risk group 53%) agreed very well with the quoted ones. Slightly worse was the compliance of the 4 year disease-free survival rates (72% in the good prognosis and 51% in the poor prognosis group) with the results from the literature. The 3 year overall survival rates (low risk patients 87%, high intermediate risk 61% and high risk patients 51%) were somewhat worse than the reported ones in all IPI subgroups except in the low intermediate risk group (82%). On the other hand, the 4 year overall survival rates of the R-IPI categories (94% in the very good prognosis group, 80% in the good prognosis group, 56% in the poor prognosis group) were much better correlated with the data from the literature.

**Conclusions:**

In total, the treatment outcomes of routinely treated patient with the DLBCL at our institute are quite encouraging when compared to results of some larger studies. There are probably no dilemmas about how to treat young good prognosis patients and patients aged over 60 years at present. However, the 5 year overall survival rate of 76% for the young poor prognosis group is unsatisfying and needs to be improved. At present, quite a few studies are underway to clarify which of the regimens will perform best in this population.

## Introduction

The diffuse large B-cell lymphoma (DLBCL) is the most common histologic subtype of non-Hodgkin’s lymphomas.^1^,^2^ However, the disease is quite heterogeneous in terms of morphology, genetics, biologic behaviour, and consequently response to treatment and prognosis.^1^ Beside histopathology and genetics, similar like in other malignancies, clinical parameters have been identified that influence the prognosis of patients with DLBCL.^3^–^5^ Namely, the age above 60 years, serum lactate dehydrogenase concentration above normal, ECOG performance status of 2 or more, Ann Arbor stage III or IV and number of involved extranodal sites above 1 have been shown to correlate significantly with a shorter disease-free and overall survival of patients treated with anthracycline containing regimen. These five factors have been included in the original international prognostic index (IPI).^3^

The addition of rituximab to standard chemotherapy (cyclophosphamide, doxorubicin, vincristine, and prednisolone [CHOP] and CHOP-like) in patients with DLBCL has resulted in significant improvements of the disease-free and overall survival rates.^6^–^8^ Beside the original IPI that has been later on validated also in patients receiving rituximab containing treatment^9^ similarly the revised international prognostic index (R-IPI)^10^ has been proposed to predict outcome in patients with the DLBCL receiving R-CHOP or R-CHOP-like regimens. It is still unclear which of the two indexes is more appropriate for presentation of study results in this population.

Due to superior results with rituximab, the R-CHOP and variants have become the standard initial treatment of patients with the DLBCL.^11^ However, the conditions of routine treatment are far different from the ones in clinical studies where the study population is highly selected, the histopathology and staging procedures are thoroughly revised and treatment and side effects are strictly controlled. The aim of our retrospective study was therefore to analyse and to compare the treatment results of routinely treated patients with the DLBCL at the Institute of Oncology Ljubljana to results reported by some larger studies.

## Patients and methods

Two hundred and ninety five patients with the DLBCL were treated between 2004 and 2008 according to the then protocol with R-CHOP or R-CHOP-like regimens at the Institute of Oncology Ljubljana. The patients’ characteristics, patohistological diagnosis, disease stage, response to treatment and survival data were taken from patients’ records. Treatment response was evaluated according to Cheson’ criteria^12^,^13^ and the disease-free and overall survival by means of Kaplan Meier survival curves. For the determination of statistical differences the log rank test and Chi-square test were applied.

## Results

### Patients’ characteristics and treatment

Among 295 patients, there were 132 males (44.7%) and 163 females (55.3%). Their median age was 64 years (range from 19 to 86 years). One hundred and sixteen patients (39.3%) were aged below 60 years and 179 patients (60.7%) were aged 60 or more years. Ninety three (31.5%) patients had limited disease (stage I or II) and 198 (67.1%) patients had advanced disease (stages III and IV) at presentation. The stage of disease could not be defined in 4 patients. According to the IPI categories, there were 34 (11.5%) patients with IPI 0, 63 (21.4%) patients with IPI 1, 66 (22.4%) patients with IPI 2, 69 (23.4%) patients with IPI 3, 46 (15.6%) patients with IPI 4 and 17 (5.7%) patients with IPI 5, respectively.

All patients were treated with R-CHOP or R-CHOP-like regimens (Table 1). The selection of regimen was influenced only by adverse prognostic factors (*e.g.* massive infiltration of bone marrow and/or bones where middle dose MTX was added to R-CHOP). In just few young poor-prognosis patients, the more dose-intensive R-ACVBP_21_ regimen was used instead of the R-CHOP_21_ regimen. The R-CHOP_14_ regimen has never been applied. In patients with compromised cardiac function, reduced doses of anthracyclines were applied and were sometimes compensated for with the addition of etoposide (reduced intensity R-CHOEP). Patients with stage I or II of the disease received 6 cycles while patients with stage I.X, II.X, III and IV received 8 cycles of rituximab containing treatment. Patients treated with R-ACVBP received 6 cycles at maximum.

### Response to treatment

The response to treatment for all patients and for distinct IPI categories is presented in Table 2. The difference in response between the low risk group and low intermediate risk group was statistically insignificant as was the difference between the high intermediate and high risk groups. But the significant Chi-square for the entire table (p=0.045) indicates the significant difference between both low risk and both high risk groups.

**FIGURE 1:**
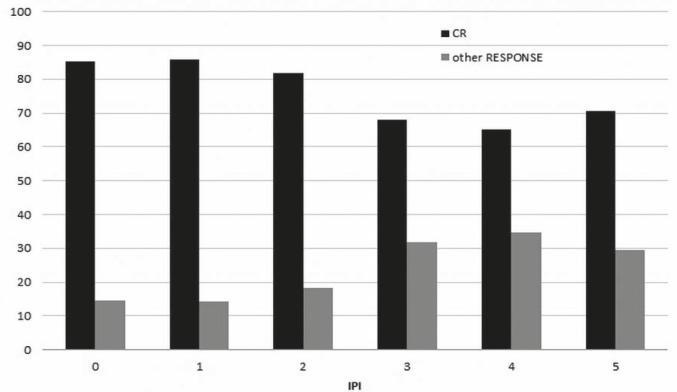
Percentage of patients achieving complete and other responses according to various IPI categories. IPI = international prognostic index. CR = complete response

The response to treatment is given also for distinct R-IPI categories (Table 3). In this case, the difference between the very good and good prognosis groups was statistically insignificant, as well as the entire table Chi-square p value (p=0,088). A statistically significant difference in the response was observed between the IPI 2 and IPI 3 categories which was detected by both indexes – namely by the IPI and the R-IPI and is also clearly presented in Figure 1.

### The disease-free survival according to IPI and R-IPI categories

**FIGURE 2:**
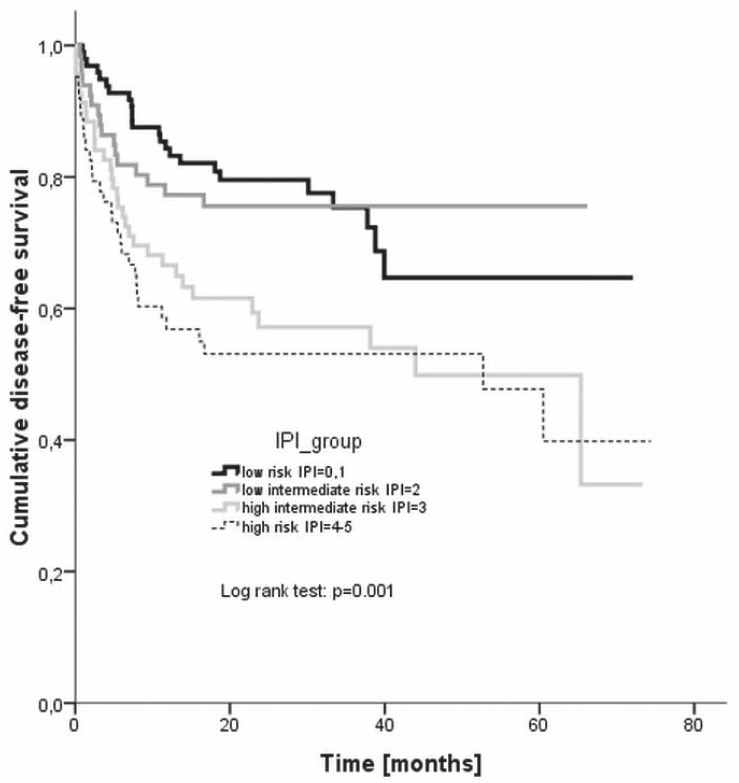
Disease-free survival according to different IPI risk groups. IPI = international prognostic index.

With the median observation period of 22 months, the estimated 3 year disease-free survival rates were 75.3% for low risk, 75.6% for low intermediate risk, 57.2% for high intermediate risk, and 53.1% for high risk group, respectively (Figure 2). The difference between the groups was statistically significant (log rank, p = 0.001).

**FIGURE 3:**
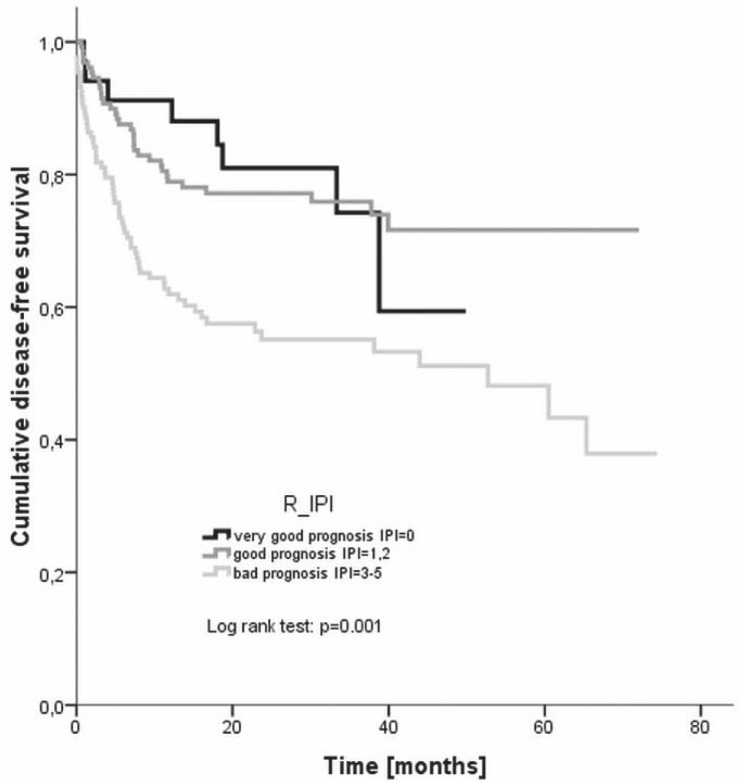
Disease-free survival according to different R-IPI risk groups. R-IPI = revised international prognostic index.

The progression-free survival was plotted also according to the R-IPI categories - the estimated 4 year disease-free survival rates were 59.4% for very good prognosis, 71.6% for good prognosis, and 51.1% for bad prognosis group, respectively (Figure 3). Again, the difference between the groups was statistically significant (log rank, p= 0.000).

### The overall survival according to IPI and R-IPI categories

**FIGURE 4:**
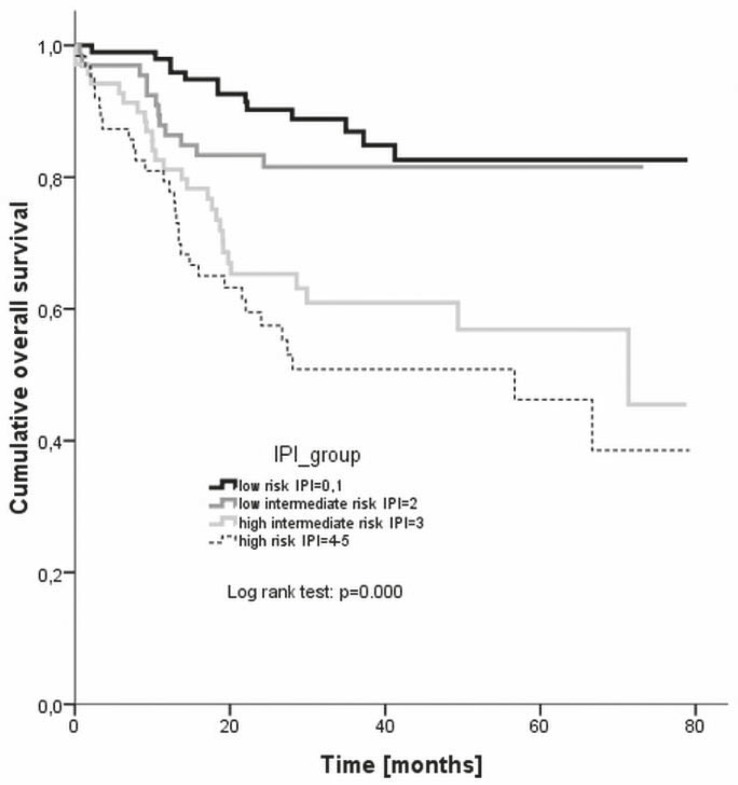
Overall survival according to different IPI risk groups. IPI = international prognostic index.

With the median observation period of 31 months, the estimated 3 year overall survival rates were 86.9% for low risk, 81.6% for low intermediate risk, 60.9% for high intermediate risk, and 50.9% for high risk group, respectively (Figure 4). The difference between the groups was statistically significant (log rank, p = 0.000).

**FIGURE 5:**
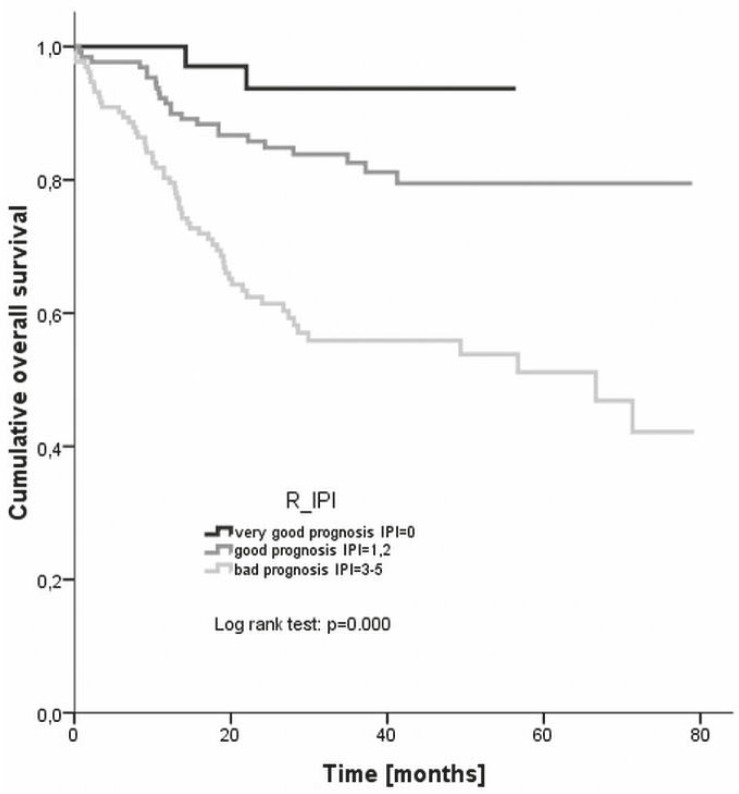
Overall survival according to different R-IPI risk groups. R-IPI = revised international prognostic index.

The overall survival was plotted also according to the R-IPI categories - the estimated 4 year overall survival rates were 93.7% for very good prognosis, 79.5% for good prognosis, and 55.9% for bad prognosis group, respectively (Figure 5). Again, the difference between the groups was statistically significant (log rank, p= 0.000).

### Treatment outcomes according to clinical categories of patients

Treatment outcomes were evaluated also according to clinical categories – namely, response to treatment, disease-free survival and overall survival were followed separately for young good prognosis patients (younger than 60 years with IPI 0 or 1), young poor prognosis patients (younger than 60 years with IPI of 2 or more), and older patients (aged over 60 years regardless of IPI), respectively.

**FIGURE 6:**
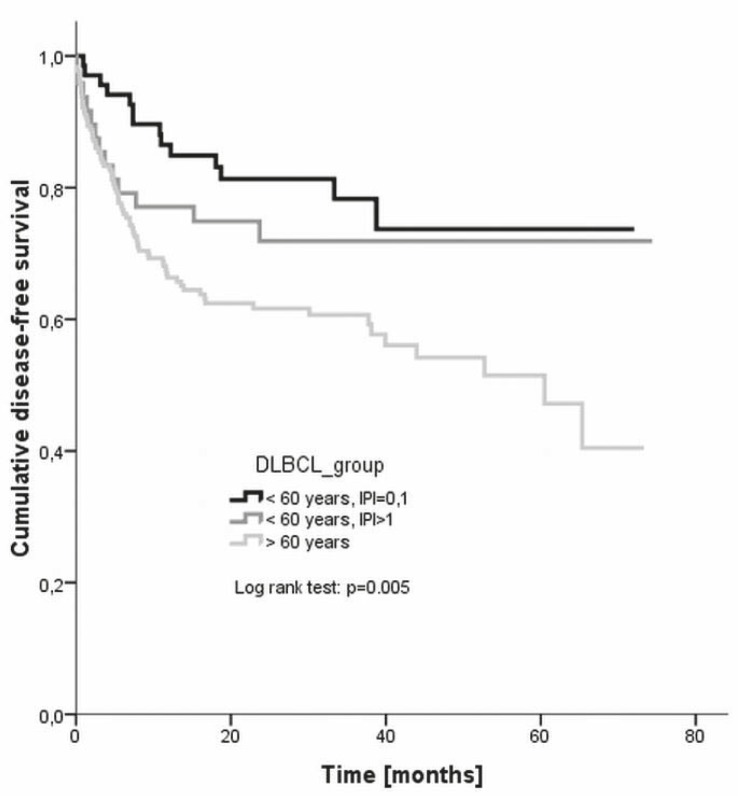
Disease-free survival of different clinical categories of patients. DLBCL = diffuse large B-cell lymphoma. IPI = international prognostic index.

**FIGURE 7:**
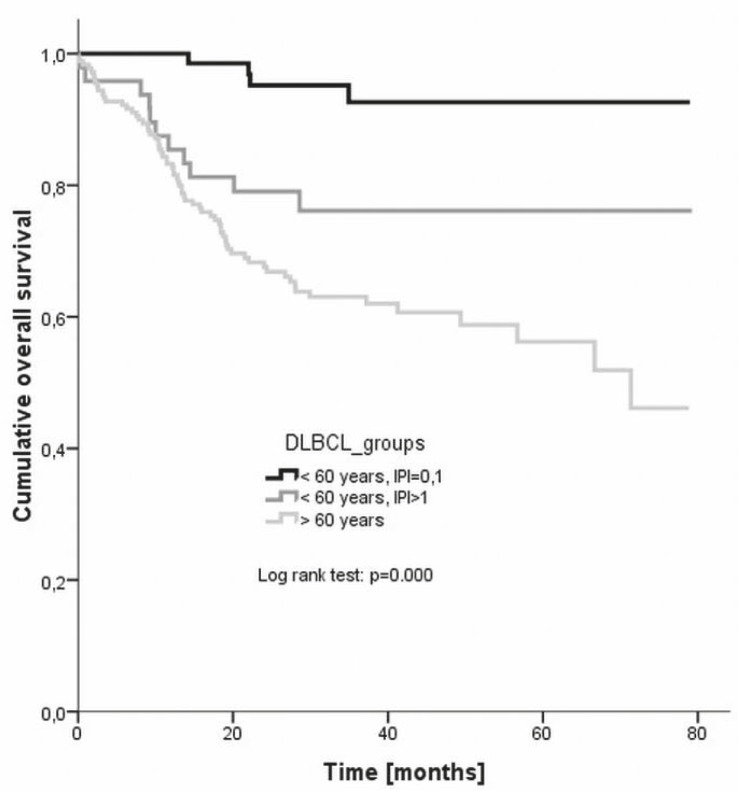
Overall survival of different clinical categories of patients. DLBCL = diffuse large B-cell lymphoma. IPI = international prognostic index

The response to treatment is given in Table 4, while the disease-free and overall survivals are plotted in Figures 6 and 7, respectively. The difference in the disease-free survival between all three groups was statistically significant (log rank, p = 0.005), but it was insignificant when only young good prognosis and young poor prognosis groups were compared (p = 0.365). Also the difference in the overall survival between all there groups was significant (p = 0.000) as was the difference between young good prognosis and young poor prognosis group (p = 0.005).

## Discussion

The treatment outcomes in patients with the DLBCL have been significantly improved with the addition of rituximab to standard anthracycline containing chemotherapies both in terms of the disease-free as well as the overall survival. This has been demonstrated by various researchers during the last decade^14^–^21^, which resulted in the introduction of rituximab into standard first-line treatment of these patients. However, it is somewhat difficult to compare the results of different studies due to variable study designs and regimens applied and therefore we are still uncertain about the optimal therapy for a given patient or for a given group of patients.^22^ Consequently, quite problematic is also the evaluation of treatment outcomes in patients treated in everyday clinical practice. The introduction of the standard IPI by Shipp *et al.*^3^, its validation in patients receiving rituximab containing treatments by Ziepert *et al.*^9^ and the proposal of R-IPI by Sehn *et al.*^10^, beside determining the patients’ prognosis at least partially facilitated the comparison of study results as well as the comparison of routine treatment outcomes with the study results.

Response to treatment in our evaluation diverged from the reported one predominately in the low risk group (CR rate of 85.6%) where it was even lower than the reported 87% CR rate in the original IPI study where patients received chemotherapy without rituximab.^3^ The same observation holds true for the very good prognosis group in the R-IPI categorisation in which a higher CR rate from the observed 85.3% could have been expected. The determined CR rates in other IPI and R-IPI groups were generally within expectations. The CR rates observed in the group of young good prognosis patients (86.8% of patients achieving CR or CRu) are completely in agreement with the results reported by Pfreundschuh *et al*. in the MInT study.^19^ However, the overall response rate of 88.3% achieved in our patients aged over 60 years was much better than the 77% overall response rate reported by Habermann *et al.*^18^

Regarding the duration of response given by the disease-free survival, again the largest discrepancy occurred in the low-risk patient group where the 3 year disease-free survival rate was 75% compared to 87% reported by Ziepert *et al*.^9^ In all other risk groups the disease-free survival at 3 years (low intermediate risk 76%, high intermediate risk group 57%, high risk group 53%, respectively) agreed very well with the reported ones (75%, 59% and 50%, respectively).^9^ An even larger discrepancy was noted in case of the R-IPI categories – namely, the 4 year disease-free survival rate was 59% in the very good prognosis, 72% in the good prognosis and 51% in the poor prognosis group, respectively, as compared to the reported 94%, 80%, and 53%, respectively.^10^ The 3 year disease-free survival of young good prognosis patients in our evaluation was 78% while Pfreundshuh *et al*.^19^ reported of 85% rate in equivalent population. On the other hand, Habermann *et al.*^18^ achieved a 53% 3 year disease-free survival rate in older patients as compared to the 61% rate in our study.

The 3 year overall survival rate of the low risk patients (87%) in our analysis was somewhat worse than the 91% reported by Ziepert *et al.*^9^ Equivalent or slightly worse were also the 3 year overall survival rates of low intermediate risk, high intermediate risk and high risk patients (82%, 61%, 51%, respectively) as compared to the reported rates (81%, 65%, 59%, respectively).^9^ On the other hand, the 4 year overall survival rates of the R-IPI categories (94% in the very good prognosis group, 80% in the good prognosis group, and 56% in the poor prognosis group, respectively) were much better correlated with the reported ones of 94%, 79%, and 55%, respectively.^10^ The 3 year overall survival of young good prognosis patients in our evaluation was 93% which completely corresponds to the rate reported by Pfreundshuh *et al*.^19^ in equivalent population. Then again, Habermann *et al.*^18^ achieved a 67% 3 year overall survival rate in older patients (aged over 60 years) as compared to the 63% rate in our study.

The repeating pattern of worst results achieved in our low risk and/or the very good prognosis group raises the question of whether those patients have been in some way “understaged”. Another possible explanation is the existence of some not yet determined aspect that negatively influenced response to treatment, disease-free survival and to some extent also the overall survival of these patients. This aspect could be of patohistological origin – *e.g*. the inclusion of patients with immunoblastic variants of the DLBCL which are no longer recognized as a separate entity in the WHO classification but have been associated with a worse outcome even after treatment with R-CHOP^23^ or of genetic origin – namely patients with the activated B-cell type gene expression profile have a much worse 5 year overall survival compared to patients with the germinal centre type gene expression profile.^24^,^25^ It is, however, quite unlikely that patients with immunoblastic lymphomas or activated B-cell type lymphomas would have been gathered prevailingly in the low risk and/or very good prognosis groups.

In total, the treatment outcomes of routinely treated patient with the DLBCL at our institute are quite encouraging when compared to results of some larger studies. There are probably no dilemmas about how to treat young good prognosis patients at present – it is with 6 cycles of R-CHOP_21_.^19^ On the other hand, for patients aged over 60 years the Ricover-60 study reported the best results with 6 cycles of R-CHOP_14_ (and total 8 applications of rituximab).^26^ This regimen is unfortunately associated with serious toxicity and therefore not applicable in the routine setting. Regarding our results also the treatment with 6 or 8 cycles (considering the stage of the disease) of R-CHOP_21_ will be appropriate for everyday management of the DLBCL in this fragile population. As for the young poor prognosis group – the 5 year overall survival rate of 76% is unsatisfying and needs to be improved. At present, quite a few studies are underway to clarify which of the regimens will perform best in this population. Most probably this will have to include routine determination of the gene expression profile in each patient in order to tailor his individual treatment.

## Figures and Tables

**TABLE 1:** Distribution of patients according to the selected regimens

	**Number**	**%**
R-ACVBP	19	6.4
R-CHOP	253	85.8
R-CHOP+MTX	12	4.1
R-CHOP/R-other	11	3.7
Total	295	100.0

R-ACVBP = rituximab, doxorubicin, cyclophosphamide, vincristine, bleomycin, prednisolone; R-CHOP = rituximab, doxorubicin, cyclophosphamide, vincristine, prednisolone; R-CHOP+MTX = rituximab, doxorubicin, cyclophosphamide, vincristine, prednisolone, middle-dose methotrexate; R-CHOP/R-other = R-CHOP with reduced doses of doxorubicin + etoposide

**TABLE 2:** Response to treatment according to different IPI categories

	**All patients**		**Low risk IPI=0,1**		**Low intermediate risk IPI=2**		**High intermediate risk IPI=3**		**High risk IPI=4–5**	

	N	%	N	%	N	%	N	%	N	%
CR	226	76,6	83	85,6	54	81,8	47	68,1	42	66,7
CRu	4	1,4	1	1,0	0	0,0	0	0,0	3	4,8
PR	36	12,2	10	10,3	4	6,1	14	20,3	8	12,7
SD	2	0,7	0	0,0	0	0,0	1	1,4	1	1,6
PD	13	4,4	1	1,0	4	6,1	4	5,8	4	6,3
Unclassified	14	4,7	2	2,1	4	6,1	3	4,3	5	7,9
Total	295	100,0	97	100,0	66	100,0	69	100,0	63	100,0

CR = complete response; CRu= complete response unconfirmed; PR = partial response; SD = stable disease; PD = progressive disease; unclassified – unclassified response to treatment; IPI = international prognostic index; N = number of patients

**TABLE 3:** Response to treatment according to different R-IPI categories

	**All patients**		**Very good prognosis IPI=0**		**Good prognosis IPI=1,2**		**Bad prognosis IPI=3–5**	

	N	%	N	%	N	%	N	%
CR	226	76,6	29	85,3	108	83,7	89	67,4
CRu	4	1,4	0	0,0	1	0,8	3	2,3
PR	36	12,2	5	14,7	9	7,0	22	16,7
SD	2	0,7	0	0,0	0	0,0	2	1,5
PD	13	4,4	0	0,0	5	3,9	8	6,1
Unclassifed	14	4,7	0	0,0	6	4,6	8	6,1
Total	295	100,0	34	100,0	129	100,0	132	100,0

CR = complete response; CRu= complete response unconfirmed; PR = partial response; SD = stable disease; PD = progressive disease; unclassified = unclassified response to treatment; R-IPI = revised international prognostic index; IPI = international prognostic index; N = number of patients

**TABLE 4:** Response to treatment according to different clinical categories. The difference among groups was insignificant (p=0.150)

	**All patients**		**<60years, IPI=0,1**		**<60years, IPI>1**		**>60years**	

	N	%	N	%	N	%	N	%
CR	226	76,6	58	85,3	34	70,8	134	74,9
CRu	4	1,4	1	1,5	0	0,0	3	1,7
PR	36	12,2	8	11,8	7	14,6	21	11,7
SD	2	0,7	0	0,0	1	2,1	1	0,6
PD	13	4,4	0	0,0	5	10,4	8	4,5
Unclassified	14	4,7	1	1,5	1	2,1	12	6,7
Total	295	100,0	68	100,0	48	100,0	179	100,0

CR = complete response; CRu= complete response unconfirmed; PR = partial response; SD = stable disease; PD = progressive disease; unclassified = unclassified response to treatment; IPI = international prognostic index; N = number of patients
